# Research and Development of a Wireless Self-Powered Sensing Device Based on Bridge Vibration Energy Collection

**DOI:** 10.3390/s21248319

**Published:** 2021-12-13

**Authors:** Xinlong Tong, Yun Hou, Yuanshuai Dong, Yanhong Zhang, Hailu Yang, Zhenyu Qian

**Affiliations:** 1 China Highway Engineering Consulting Group Company Ltd., Beijing 100089, China; zzkjhouy@vip.126.com (Y.H.); dys_bj@163.com (Y.D.); zyh03050@163.com (Y.Z.); qianzhy@hotmail.com (Z.Q.); 2 Research and Development Center on Highway Pavement Maintenance Technology, China Communications Construction Company Limited, Beijing 100089, China; 3 Research and Development Center of Transport Industry of Technologies, Materials and Equipment of Highway Construction and Maintenance, Beijing 100089, China; 4 National Center for Materials Service Safety, University of Science and Technology Beijing, Beijing 100083, China; yanghailu@ustb.edu.cn

**Keywords:** PCB, triangular, sensor, self-powered

## Abstract

Traditional bridge monitoring has found it difficult to meet the current diversified needs, and frequent replacement of sensor batteries is neither economical nor environmentally friendly. This paper presents a wireless acceleration sensor with low power consumption and high sensitivity through integrated circuit design, data acquisition and wireless communication design, package design, etc. The accuracy of the sensor in data collection was verified through calibration and performance comparison tests. The ability of triangular piezoelectric cantilever beam (PCB) was tested through design and physical manufacture. Finally, the self-powered performance of the sensor was tested by connecting the sensor and the triangular PCB through a circuit, which verifies the feasibility of using the PCB to collect bridge vibration energy and convert it into electrical energy to supply power for sensor, and also explore the green energy collection and application.

## 1. Introduction

With the rapid development of microelectronics, the sensors used in bridge health monitoring are trending towards low power consumption, low cost, small size and wireless communication [1]. At present, some scholars still use wiring to supply power when monitoring bridges, but the cost is quite expensive, and it consumes manpower and financial resources. The power supply using chemical batteries also needs to be replaced regularly. The power consumption of sensors in the health monitoring system has become a more concerning issue for bridge managers [2]. Especially for the current rapid development of wireless networks and embedded systems, battery-powered defects are more obvious. The global primary energy supply is based on the predictable depletion of petrochemical energy. There is an urgent need to accelerate the development of renewable energy to provide power for these low-energy electronic products, which has become an urgent problem to be solved [3,4].

Vehicles running on the bridge will produce vibration, and the driving load can will cause stress, strain and displacement to the bridge structure. The bridge will obtain vibration energy from the driving load and gravity, which will save a lot of energy if this vibration energy can be utilized [5,6].

Piezoelectric technology is a kind of energy conversion technology [7,8]. Pulkit Sharma has studied the sintered Ga-modified PZT ceramics and analyzed impedance and modulus characteristics indicated on non-Debye relaxation and significant contributions of grains on electrical properties [9]. Manisha Sahu has studied energy harvesting performance in a triboelectric nanogenerator using ferroelectric polarization for self-powered IR signaling and body activity monitoring, the performance of the device is to design a multi-stack hybrid generator (MS-HG) that could generate an electrical output of 300 V and a power density of 157 mW m^−2^ upon mechanical excitation [10].

PCB is a kind of power generation structure used in a vibration environment, which vibrates along with the environment, and it can produce the highest power output when resonance is reached [11,12,13]. Liu Yinan studied the structure of the piezoelectric cantilever beam, and a rectangular piezoelectric cantilever beam structure was designed and optimized by combining the theoretical calculation and the simulation [14]. The natural frequency of the piezoelectric cantilever beam was 8.83 Hz and the voltage extreme value reached 37 V through experiments. In order to improve the energy conversion performance of a piezoelectric cantilever beam energy harvester (PCEH), Xiong Y put forward a novel PCEH according to the typical PCEH [15]. The results show that the first-order eigenfrequency of the novel PCEH is 43.7 Hz and the optimal output power is 10.69 mW. Uddin designed and simulated a PCB for energy harvesting using mechanical ambient vibration. The PCB consisted of a copper substrate, two piezoelectric layers, and a base. In the mechanical properties analysis, a maximum output power of 14.85 μW and voltage of 595.5 mV was obtained from the harvester at 12.6 kΩ under the acceleration of 1 g (g = 9.81 m/s^2^) at a resonant frequency of 345.75 Hz. This energy harvester can be used for numerous purposes in the field of sensors and wireless sensor networks.

If the PCB is placed on the bridge, then it can absorb the vibration energy of the bridge and convert it into electrical energy to supply power to the sensor or indicator light [16]. However, the bridge vibration frequency is low, and the piezoelectric cantilever beam used for bridge vibration energy collection must have a very low resonance frequency and high performance output. This paper has studied the principle of wireless sensors, and a wireless acceleration sensor was developed based on the needs of bridge health monitoring, which can be conveniently placed anywhere on the bridge. Then, the structure of the PCB is studied and optimized to improve the piezoelectric output performance according to the characteristics of the bridge vibration. Finally, a piezoelectric transducer device suitable for bridge vibration energy collection and wireless sensor power supply is developed, which realizes the self-powered performance of bridge sensor nodes. The voltage of the triangular PCB developed in this paper reaches the first peak value of 51.6 V at 3.2 Hz, and the voltage reaches the second peak value of 77.2 V at 12.1 Hz; its resonance frequency is lower than that of the cantilever beam developed by previous scholars, and the output performance is higher, which is more suitable for application in the bridge’s low frequency vibration environment.

## 2. Wireless Acceleration Sensor

Sensors are the node for data connection in bridge health monitoring [17]. A wireless acceleration sensor was developed through research on wireless communication technology in this paper, the development of circuit boards, design and manufacture of enclosures, etc. The sensor has a certain computing capability, which can extract characteristic indexes such as peak acceleration and vibration frequency, and reduce communication energy consumption; furthermore, it can improve data transmission stability and efficiency, which is suitable for bridge vibration monitoring [18].

### 2.1. The Composition of Acceleration Sensor

#### 2.1.1. Circuit Diagram

**Figure 1:**
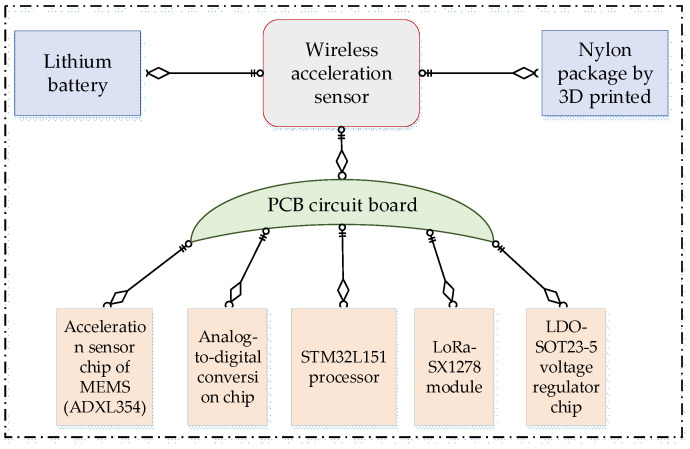
The composition of wireless acceleration sensor.

The overall structure of the wireless acceleration sensor is composed of a PCB circuit board, lithium battery, and 3D-printed nylon package, as shown in Figure 1. The PCB board is mainly composed of circuits modules, resistors, and capacitors to ensure mutual communication between the modules. The modules are an MEMS acceleration sensor chip ADXL354, AD7689 analog-to-digital conversion chip, STML32L151C8x6 processor, LoRa-SX1278 wireless communication module and LDO-SOT23-5 voltage regulator. The data collected by the acceleration chip are converted into a digital signal by the AD7689 chip, and then processed by the STML32L151C8X6 processor. Finally, the data are wirelessly transmitted to the gateway through the LoRa-SX1278 module [19,20] and can also be transmitted to the computer through the serial port.

The wireless acceleration sensor uses a 3D nylon printed package design on the package, which has the advantages of more robustness, toughness, and impact resistance, and it is waterproof. The circuit diagram of the acceleration sensor in this paper is mainly composed of the following parts.

**Figure 2:**
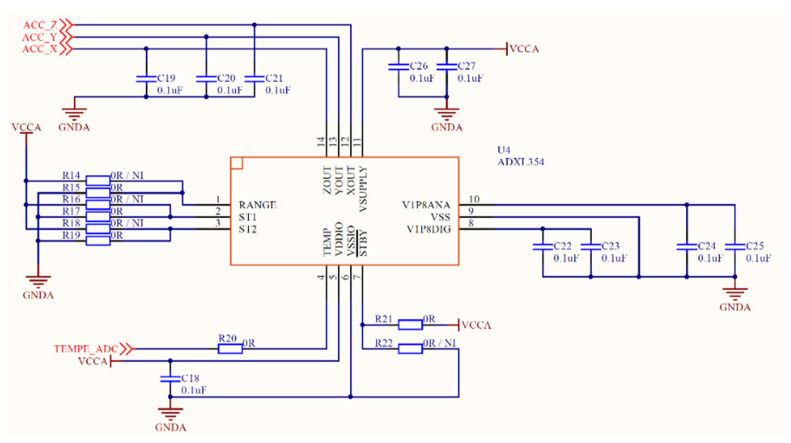
The circuit diagram of ADXL354.

The circuit diagram of ADXL354 is shown in Figure 2. It uses multiple 0.1 μF ceramic capacitors to fully decouple the accelerometer to eliminate power noise. There are high-frequency noises generated by vehicles, instruments, collisions, etc., when the sensor collects data on the bridge; therefore, the output terminals of the X, Y, and Z axes are connected with a 0.1 μF filter capacitor respectively to filter out the high signal generated by noise [21], so as to collect the raw data of bridge vibration.

**Figure 3:**
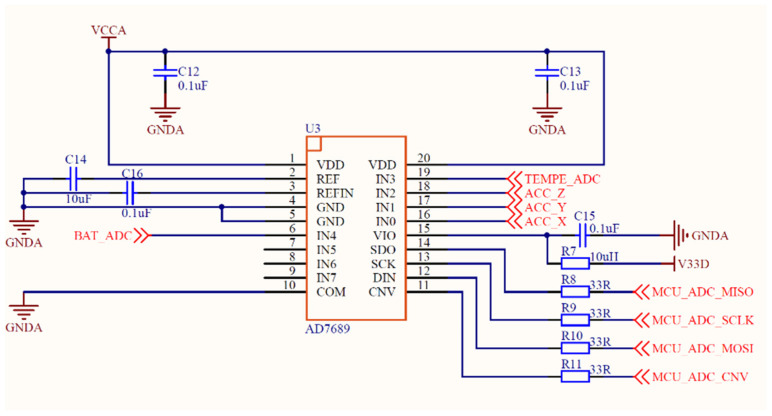
The circuit diagram of AD7689.

The circuit diagram of AD7689 is shown in Figure 3, and the data communication of AD7689 is controlled by CPU through an SPI (Serial Peripheral Interface) bus. The VDD is the working voltage inside the device, which is connected to an external power supply with a 0.1 μF decoupling capacitor in parallel. REF and REFIN are connected with 10 μF and 0.1 μF decoupling capacitors, respectively, which ensures that the AD7689 has a relatively stable power supply voltage and has an anti-interference effect.

**Figure 4:**
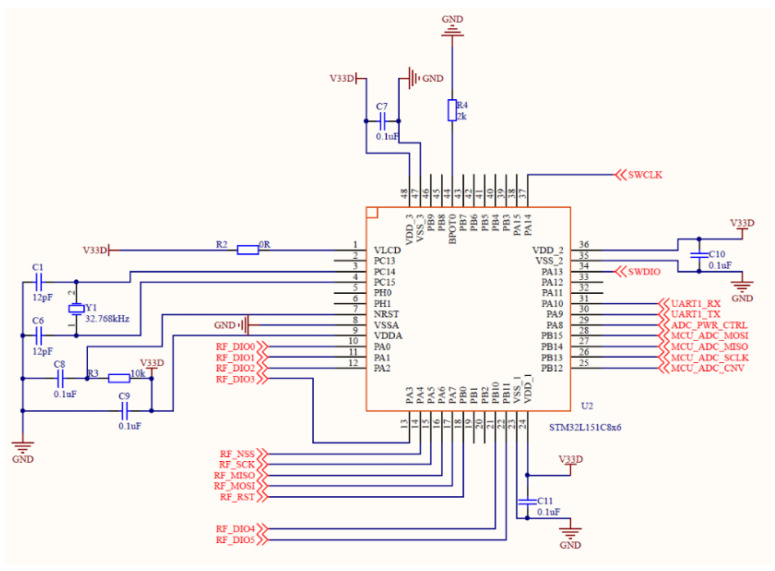
The circuit diagram of STM32L151C8X6.

STM32L151 is an ARM-based 32-bit microcontroller, as shown in Figure 4, which is an ultra-low power chip with 0.3 μA standby mode (three wake-up pins), and its core is ARM Codex-M3 CPU. The supply voltage is 2.0 V to 3.6 V. STM32L151 embeds a built-in boost converter. VDD is an external power supply for I/O and internal regulators. The minimum voltage applied to VDDA is 1.8 V when the ADC module is used. At this time, VDDA and VSSA must be connected to VDD and VSS, respectively [22].

**Figure 5:**
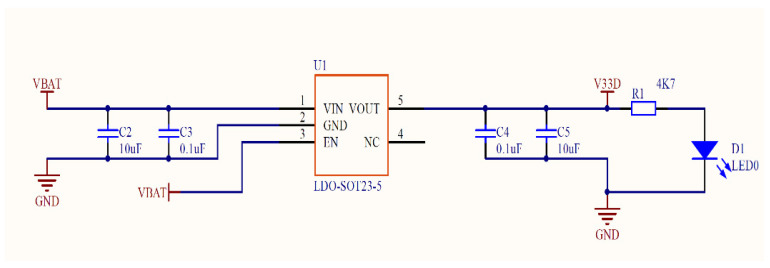
The circuit diagram of LDO-SOT23-5.

LDO-SOT23-5 is a voltage regulator chip which reduces the battery voltage output to 3.3 V through the acquisition circuit and continuously supplies power to the CPU. The resistor R1 (4.7 kΩ) can play the role of current limiting to protect the circuit, as shown in Figure 5.

**Figure 6:**
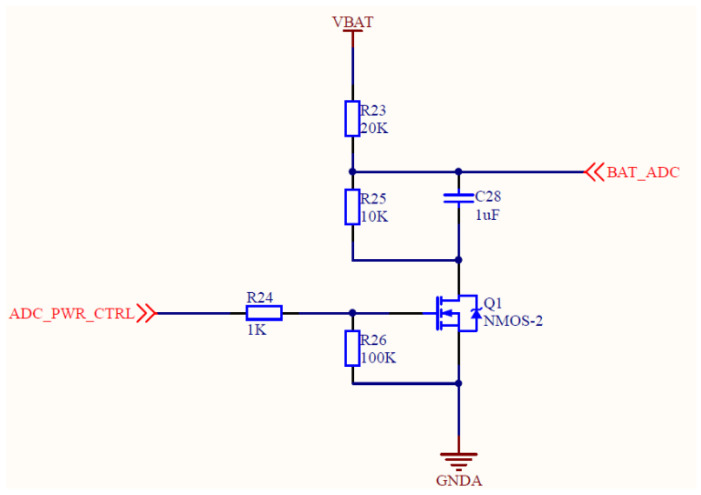
The circuit diagram of battery voltage acquisition circuit.

**Figure 7:**
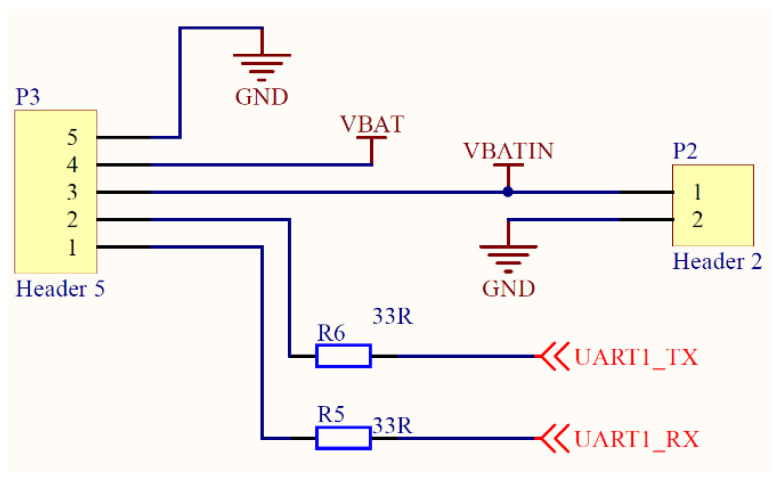
The circuit diagram of serial port and power interface.

A battery voltage acquisition circuit is shown in Figure 6. The sensor collects the voltage of the lithium battery through this circuit, and then reduces the battery voltage to 3.3 V through the LDO-SOT23-5 circuit to supply power to the CPU. The circuit diagram of a serial port and power interface is shown in Figure 7. The serial port can output collected data to the computer or supply power to the lithium battery by connecting an external power.

#### 2.1.2. Production and Package of Circuit Board

**Figure 8:**
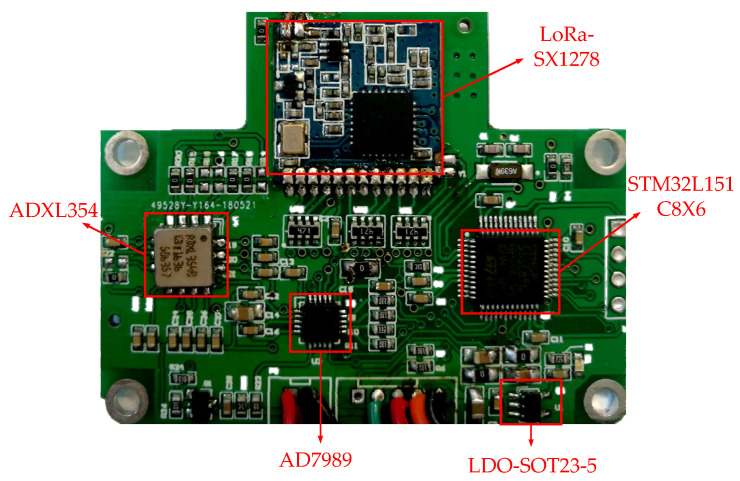
Circuit board of acceleration sensor.

In this paper, the circuit board and package were made according to the composition structure of the wireless acceleration sensor and the circuit diagram. The PCB circuit board is shown in Figure 8.

The red boxes in Figure 8 are an ADXL354 accelerometer chip, STM32L151C8X6 processing, AD7989 analog-to-digital conversion module, LDO voltage regulator chip, and LoRa wireless communication module. These components enable bridge vibration data to be collected in real time and transmitted wirelessly. The holes in the four corners under the circuit board are screw holes, whose purpose is to fix the circuit board to the package box and prevent the circuit board from effecting the data collection due to unstable movement.

**Figure 9:**
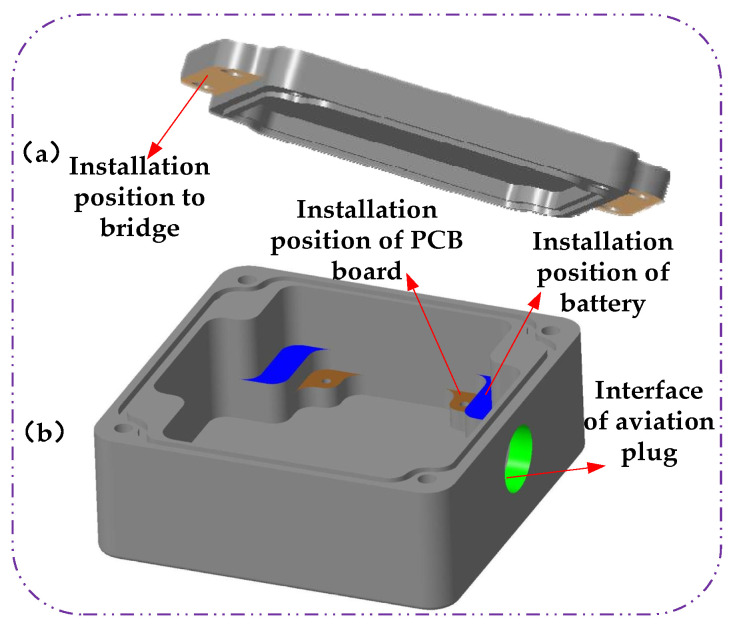
Schematic diagram of the packaging box of wireless acceleration sensor.

The package box of the wireless acceleration sensor in this paper is shown in Figure 9; (a) is the top cover of the package box. There are two outrigging ears on both sides of the top cover, whose purpose is to facilitate the installation of the sensor on the bridge. (b) is the base of the package box, the brown color in Figure 9 is the installation position of the sensor PCB board which is fixed by screws, and the blue part is the installation position of the lithium battery. The green round hole is the aviation socket. The internal lithium battery can be charged through the aviation socket, so that the sensor can be used continuously. The sensor can also be connected to the serial port USB to transmit data to the computer through the aviation socket.

**Figure 10:**
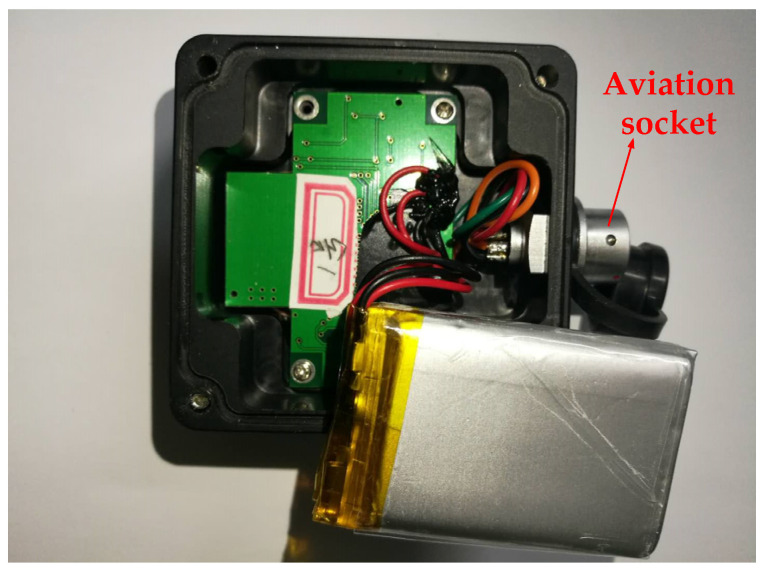
Internal view of sensor’s case before glue filling.

The package box by 3D printing was accomplished to facilitate installation of the cantilever sensor, and the material of box is nylon. The circuit board and battery were installed into the package box, as shown in Figure 10. The red arrow shows the aviation socket, which has three main functions: (1) it can be connected to the power supply to supply power for the sensor; (2) the sensor circuit board will power on and start working after being inserted into the aviation plug; (3) the data can be transmitted to the computer through the serial port.

**Figure 11:**
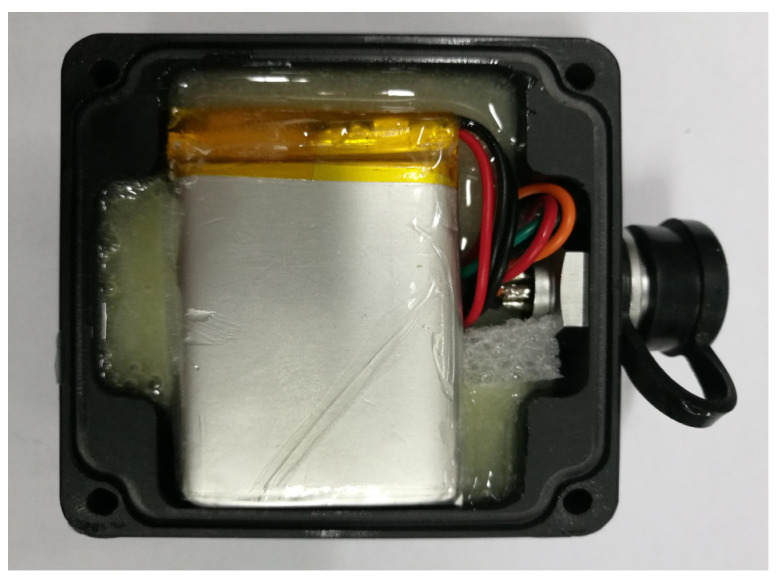
Internal view of sensor’s case after glue filling.

**Figure 12:**
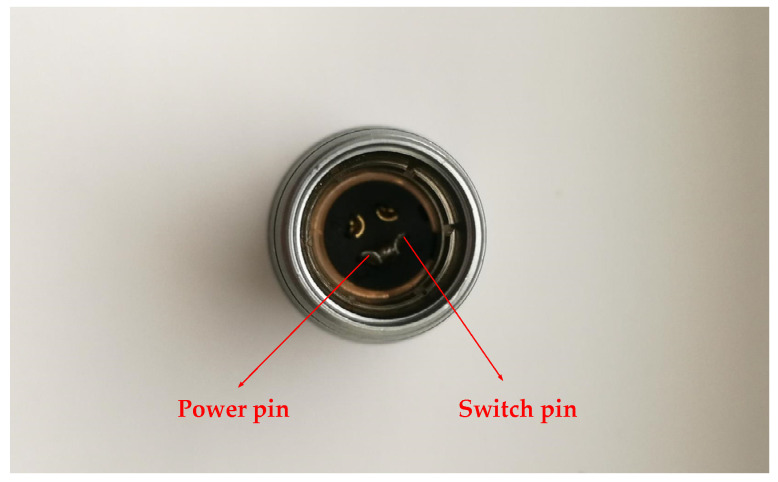
The welded of power pin with switch pin.

**Figure 13:**
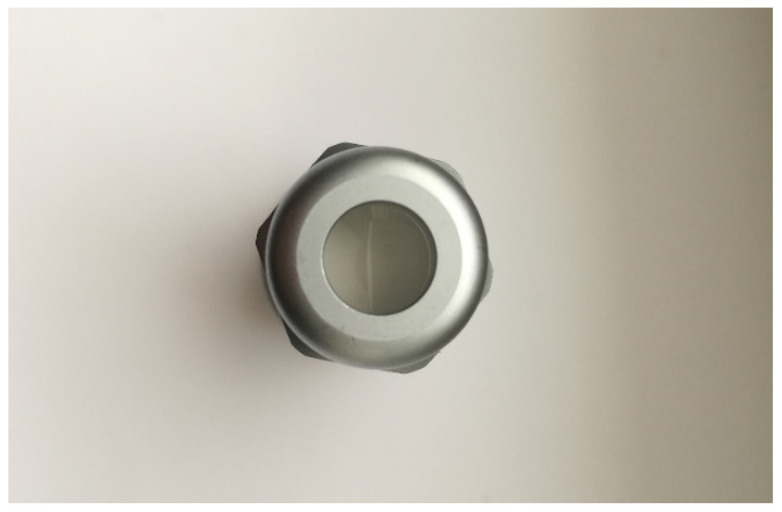
Aviation plug after glue filling.

**Figure 14:**
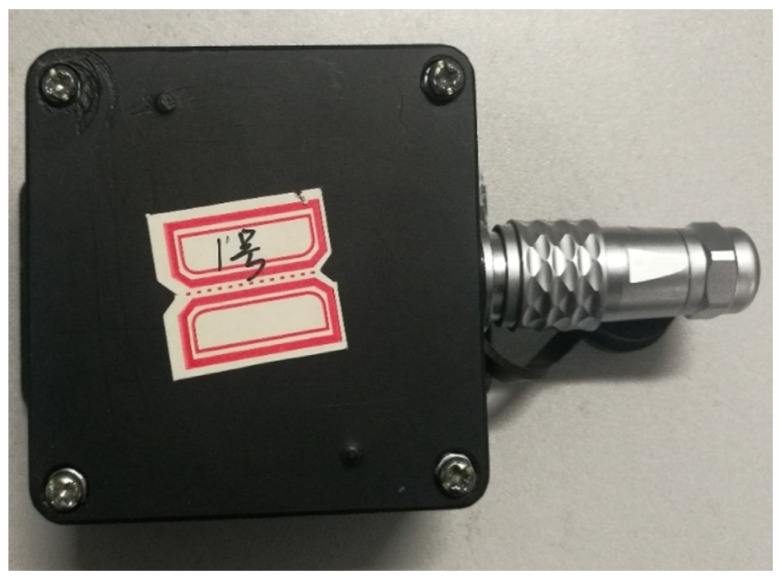
The wireless acceleration sensor.

Figure 11 shows the internal diagram of the acceleration sensor after glue filling. The circuit board and the lithium battery were fixed inside the nylon box to prevent the damage of the circuit board due to the vibration of the lithium battery. The aviation sockets of the wireless acceleration sensor developed in this paper has four pins, which are the GND ground pin, the serial output pin, the power pin and the switch pin. The aviation socket was equipped with an aviation plug which has four identical pins. The power pin and switch pin of the aviation plug were welded in this paper, and the aviation plug was filled with glue, as shown in Figure 12 and Figure 13. The wireless acceleration sensor can be connected to the power supply to collect data by inserting the aviation plug into the aviation socket, and the aviation plug after filling can also play a role in waterproofing. The finished product of the wireless acceleration sensor is shown in Figure 14.

### 2.2. Calibration of Acceleration Sensor

**Figure 15:**
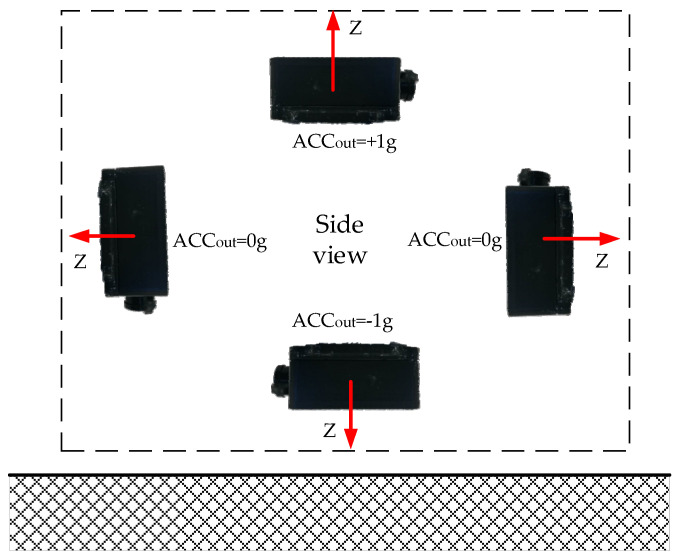
Schematic diagram of the sensor being accelerated by gravity.

Four wireless acceleration sensors have been produced in this paper which should be calibrated first. Set the three directions of the acceleration sensor as X, Y, and Z, respectively. Place the developed sensor No. 1 horizontally, with the Z direction facing upward. Its value was 34,325, and at this time, its gravity acceleration was 1 g. Place the sensor vertically, with Z direction facing horizontally. Its value was 23,898 with under 0 g of gravitational acceleration. Place the sensor horizontally in the opposite direction, with the Z direction facing downwards. Its value was 13,610. The schematic diagram is shown in Figure 15.

**Figure 16:**
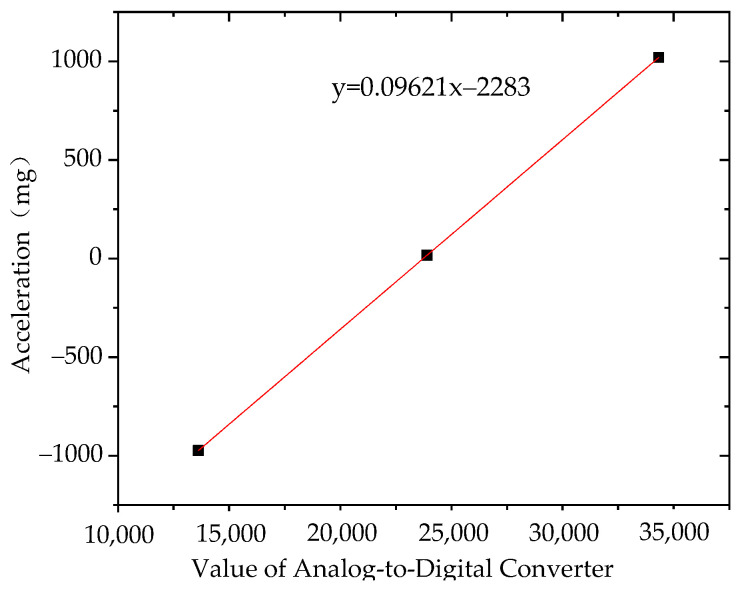
Sensor No. 1.

**Figure 17:**
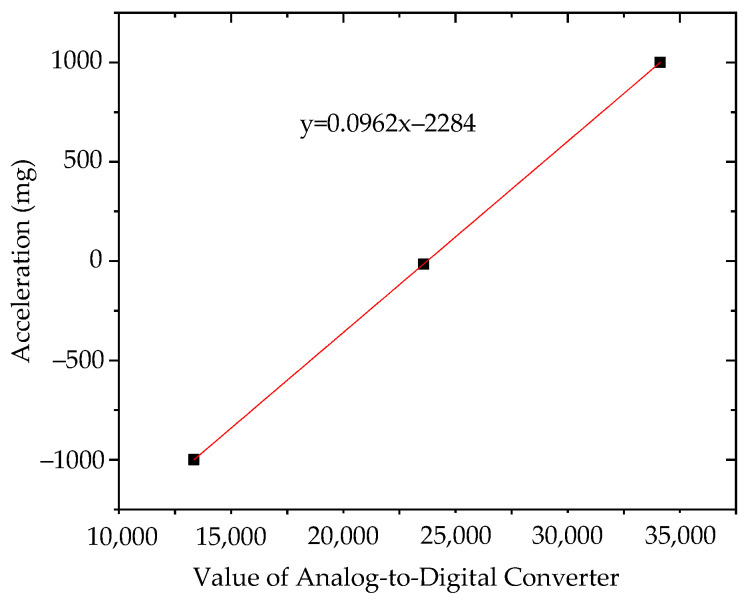
Sensor No. 2.

Fitting the analog value to its corresponding acceleration value, the results of sensor No. 1 and No. 2 are shown in Figure 16 and Figure 17.

It can be seen from the above diagram that the calculation coefficient of sensor No. 1 was 0.09621 and the intercept was 2283. The calculation coefficient of sensor No. 2 was 0.0962 and the intercept was 2284. Embed the sensitivity and intercept algorithm into the sensor; then, when the sensor is working, the converted value of the analog value will be multiplied by the calculation coefficient and the intercept is subtracted to obtain the acceleration value.

## 3. Vibration Energy Harvesting Device

Piezoelectric materials can generate electricity when subjected to external forces. In practice, this is realized by utilizing a piezoelectric transducer. PCB is a type of piezoelectric transducer structure, which has the characteristics of high flexibility, easy packaging, and low natural frequency [23]. It is suitable for transportation infrastructure and other low frequency environments. If a PCB is placed on a bridge, it will vibrate along with the bridge. The vibrational mechanical energy of the bridge caused by passing vehicles can be collected and converted into electric energy, which can supply power to the wireless sensing system.

**Figure 18:**
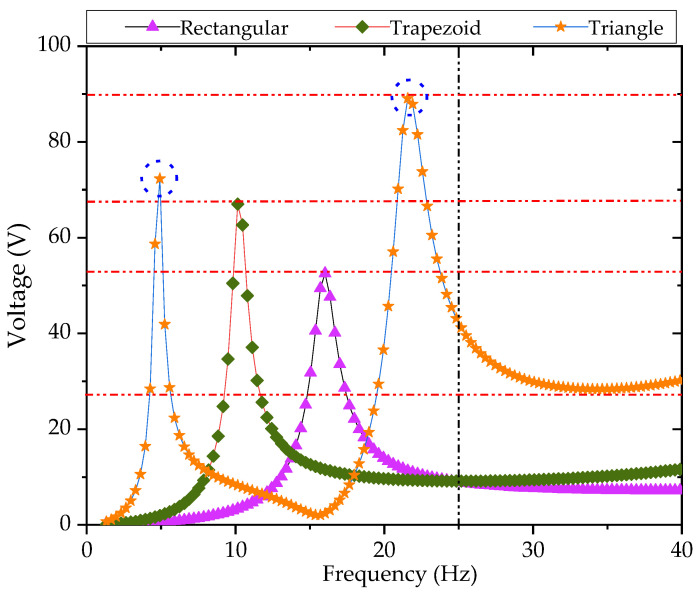
Simulation data of rectangular, trapezoidal, and triangular piezoelectrics [24].

In this paper, the author has carried out a comparative study of three different previously identified PCBs, namely rectangular, trapezoidal and triangular PCBs. The three PCBs have the same volume of thickness of the piezoelectric plate, thickness of the substrate and weight of the mass block. It has been proven that triangular is better than rectangular PCB and trapezoidal PCB [24]; not only is the vibration frequency lower than rectangular and trapezoidal cantilever beam, but also the voltage output is higher than the rectangular and trapezoidal cantilever beam, which is more suitable for the low-frequency vibration energy collection of the bridge. The generated voltage of three types of PCBs by simulation is shown in Figure 18.

**Figure 19:**
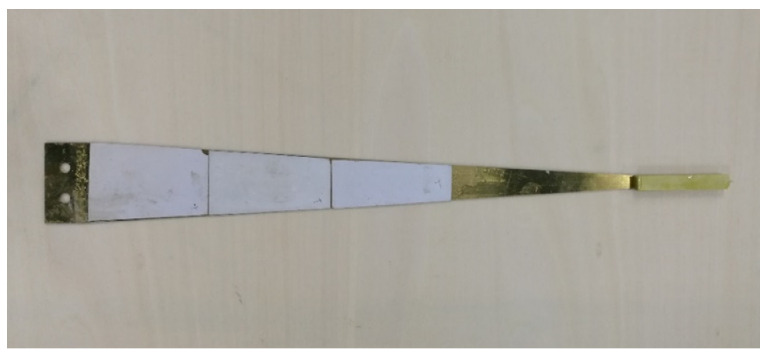
The photo of triangular PCB.

The sample of the triangular PCB was made according to the size parameters [24]: the length of the substrate is 320 mm, the width is 40 mm, and the thickness is 0.35 mm. The thickness of the piezoelectric plate is 0.25 mm and the weight of the mass block is 12 g. The sample is shown in Figure 19.

**Figure 20:**
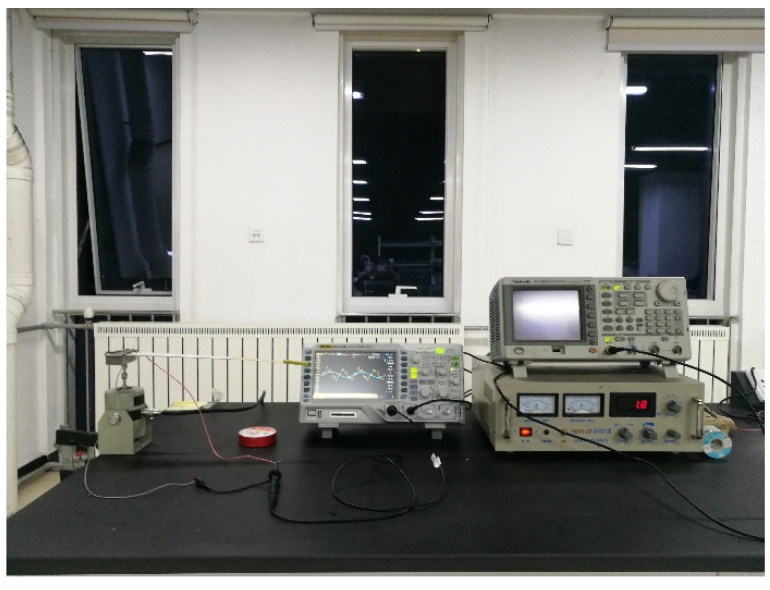
The test of triangular PCB.

**Figure 21:**
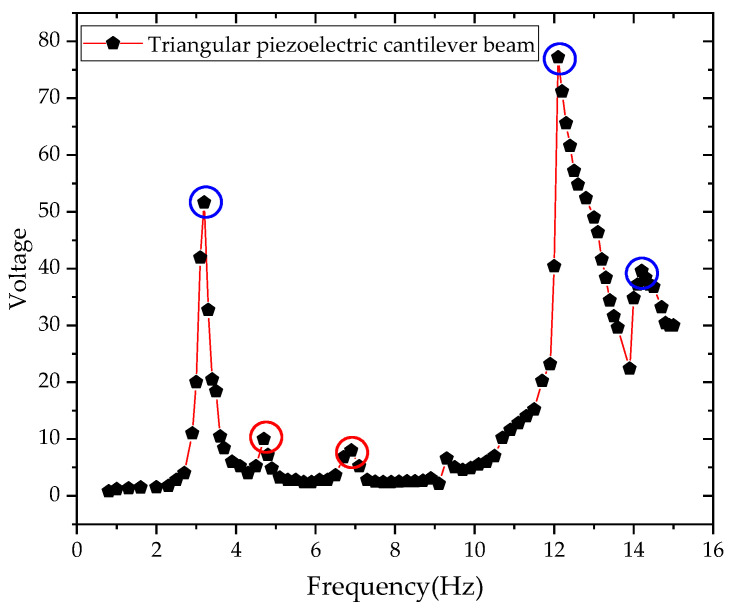
The relation between voltage and frequency of triangular PCB.

A power amplifier and a hev-20 high energy exciter were used to test the power generation capacity of a triangular PCB. Adjust the power amplifier to increase the frequency slowly to make the cantilever start to vibrate until it reaches resonance, as shown in Figure 20. The capacitor voltage value tested by the oscilloscope and the corresponding frequency tested by the power amplifier were recorded, and the relationship curve between voltage and frequency was plotted, as shown in Figure 21.

**Figure 22:**
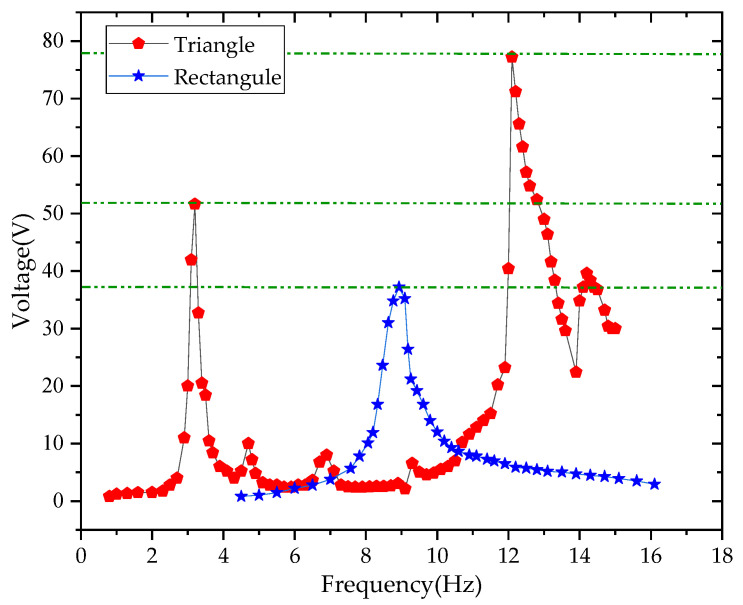
The physical test results of triangular and rectangular PCBs.

It can be seen from Figure 21 that the first- and second-order vibration frequencies of the triangular PCB were within the low frequency range (0–15 Hz). The sample reaches the first-order resonance at 3.2 Hz and a voltage value of 51.6 V. The sample reaches the second-order resonance at 12.1 Hz and a voltage value of 77.2 V. This paper has compared the physical test results of triangular and rectangular PCBs, as shown in Figure 22.

It can be seen from Figure 22 that the output voltage of the triangular cantilever beam was higher than the rectangular cantilever beam; the resonance frequency is lower, and there are more resonance points in the low-frequency range than the rectangular cantilever beam. Therefore, triangular PCBs are more practical than rectangular in terms of energy collection.

## 4. The Performance Test of Self-Power Sensor

### 4.1. The Capacity Test of Piezoelectric Power Generation

**Figure 23:**
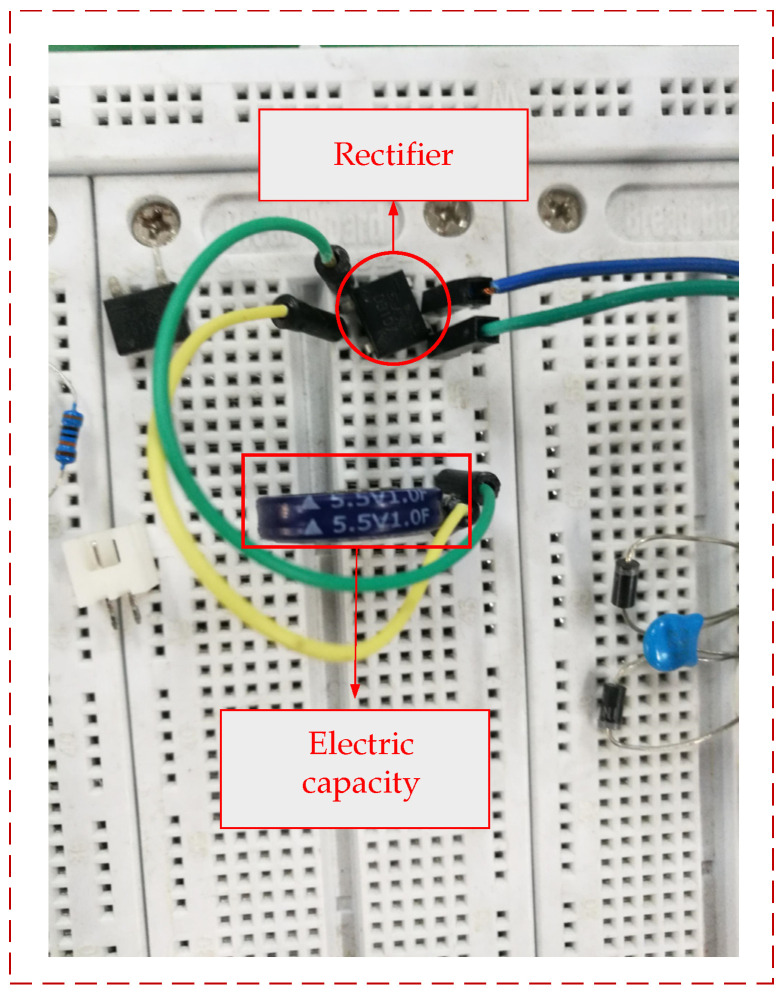
Circuit connection on breadboard.

**Figure 24:**
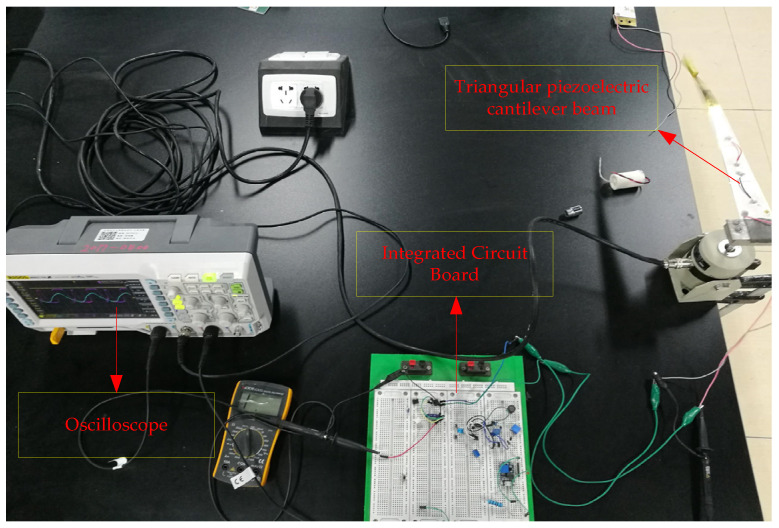
Power generation capacity test of triangular PCB.

**Figure 25:**
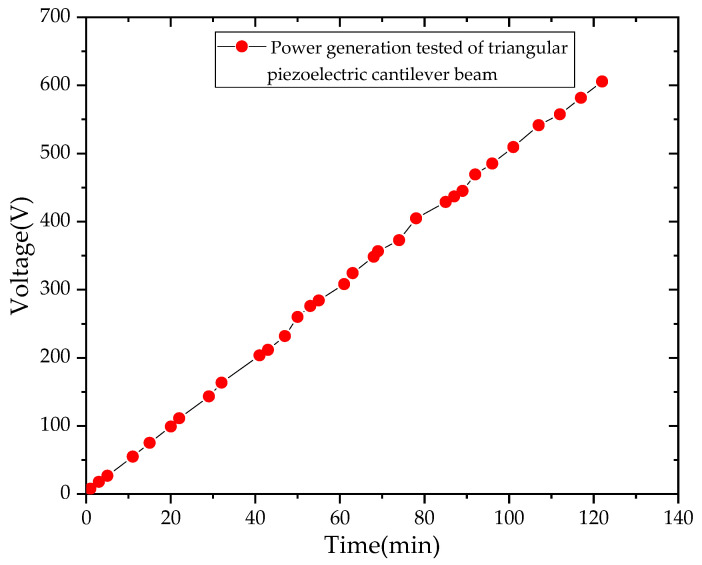
The relationship between power generated by triangular PCB with time.

The power generation capacity of triangular PCB was carried out in this paper. A power amplifier, hev-20 high energy exciter, dpo-2024 oscilloscope, breadboard and rectifying device were used to test the triangular PCB. Rectify the output voltage of the PCB on the breadboard, and then connect the capacitor for charging, as shown in Figure 23. We adjust the high energy exciter so that the excitation frequency of the cantilever beam was 12 Hz, and then connect the cantilever beam output end to the input end of the breadboard and connect the output end of the breadboard to the oscilloscope, as shown in Figure 24. The capacitor voltage value and the corresponding times tested by the oscilloscope were recorded and the relationship curve between voltage and times was plotted, as shown in Figure 25.

The final value of the voltage in the capacitor was 1.4 V through 4 h continuous collection. The relationship between electric energy and voltage can be expressed by Equation (1).

(1) $$E=\frac{1}{2}CU^{2}$$

where *C* is piezoelectric equivalent capacitance and *U* is open circuit voltage.

The power generation of the triangular PCB was 0.98 J in 4 h by Equation (1), so the power generation was 0.245 J in 1 h. The wireless acceleration sensor in this paper has a current of 28.15 mA when transmitting data. The consuming is 100 ms and the working voltage is 3.3 V, so the required electric energy was 0.009 J. A piezoelectric energy harvesting device can install eight triangular PCBs which were designed in this paper, so it can generate 1.96 J in 1 h, which can be used for the wireless acceleration sensor to transmit data 217 times.

### 4.2. The Self-Powered Performance Test of Sensor

**Figure 26:**
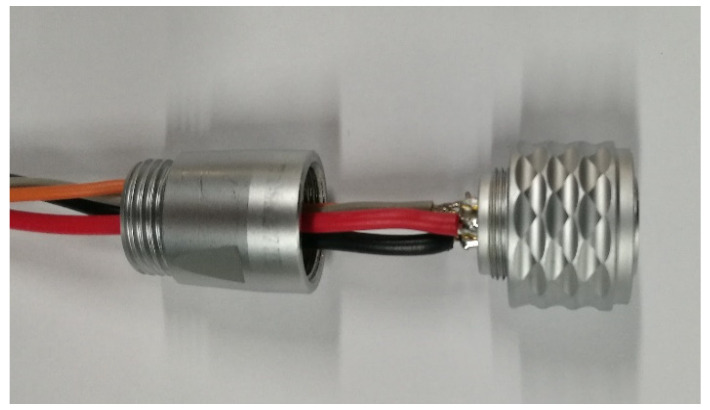
Pin circuit welding of aviation plug.

In order to test the effect in practical applications, a self-powered sensor experiment in the laboratory was carried out in this paper. There are four pins at the aviation plug of the wireless acceleration sensor in this paper, namely the power supply pin, the switch pin, the GND ground pin, and the serial output pin. The power supply pin and switch pin were connected to the capacitor, and the GND ground pin and the serial output pin were connected to the USB serial chip, as shown in Figure 26. We can download the data from the computer by connecting the USB serial chip to the computer. Additionally, the power consumption of transmitting data through the serial port is 14 mA, which is lower than the wireless transmission. The time for data transmission is 50 ms, which is less than wireless transmission.

**Figure 27:**
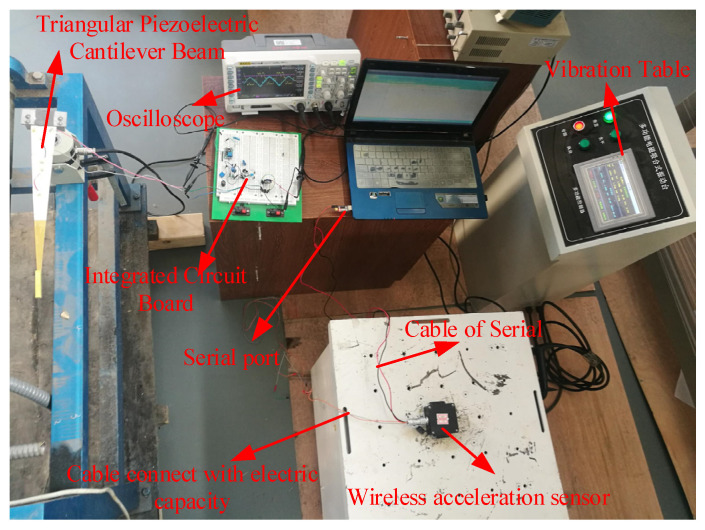
The self-powered performance test of sensor.

In this experiment, a triangular PCB was used as the source of power generation. The output power of the power amplifier was adjusted to keep the cantilever vibration frequency at 11 Hz and the fixed end amplitude at 1 mm. Fix the sensor on the vibrating table, then adjust the host parameters (frequency and strength) of the vibration table to give the vibration table a sinusoidal excitation, as shown in Figure 27. The cantilever beam continues to vibrate and generate electricity to charge for the capacitor. When the battery is charged enough for the sensor to collect and transmit data once, the sensor will start the CPU.

**Figure 28:**
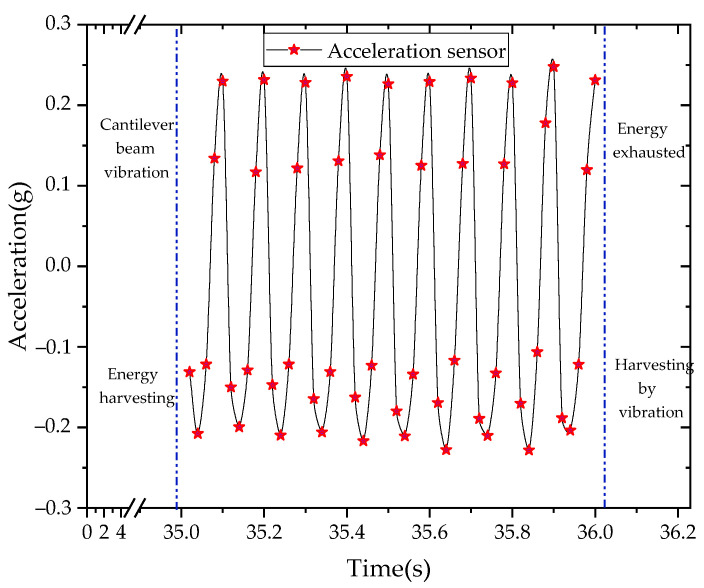
Acceleration sensor data.

We set the time when the cantilever beam starts to vibrate as the start time, and the serial interface on the computer received the sensor’s data on the vibration after the cantilever beam was vibrating for about 35 s, as shown in Figure 28.

The experiment’s results proved the effectiveness of the self-powered sensor designed in this paper. This is a preliminary research study of a self-powered sensor. Additionally, an energy management circuit will be designed and optimized to improve the energy harvesting performance of the PCB.

The dimensions of the vibration energy harvesting device does not correlate with the dimensions of the sensor. We will design and make a nylon package box to integrate the sensor and piezoelectric device and test its performance in future study.

## 5. Summary

This paper developed a wireless acceleration sensor that is suitable for bridge vibration monitoring, which is the front-end sensing node of the bridge monitoring system. Additionally, a new structure type of a PCB energy harvesting device was simulated and physically tested, which realizes the technical method of bridge piezoelectric energy harvesting. In particular, the main contributions can be summarized as follows:

1. A wireless acceleration sensor with low power consumption and high sensitivity for bridge vibration monitoring was developed, which solves the difficulties of traditional wiring monitoring. Calibration and performance comparison tests have verified the accuracy of the sensor in data collection.
2. A triangular PCB was designed and fabricated based on the research results of different shapes of PCBs. It was shown that the triangular PCB has greater power generation capacity than the rectangular and trapezoidal PCBs, and the natural frequency is lower than the rectangular and trapezoidal PCBs.
3. The acceleration sensor was integrated with the triangular PCB through the circuit, and its self-powered ability was tested. The experiment results showed that the single triangular PCB can generate 0.245 J within 1 h under excitation of 12 Hz, which proved the feasibility of installing piezoelectric energy harvesting devices on bridges to supply power for the sensors.

## Data Availability

Not applicable.

## References

[1] Xinlong T., Hailu Y., Linbing W., Yinghao M. (2019). The development and field evaluation of an IoT system of low-power vibration for bridge health monitoring. Sensors.

[2] Cassandras C.G., Wang T., Pourazarm S. (2014). Optimal routing and energy allocation for lifetime maximization of wireless sensor networks with Nonideal batteries. IEEE Trans. Control Netw. Syst..

[3] Yang H., Cao D. (2017). An investigation on stress distribution effect on multi-piezoelectric energy harvesters. Front. Struct. Civ. Eng..

[4] Kempton W., Tomi J. (2005). Vehicle-to-grid power implementation: From stabilizing the grid to supporting large-scale renewable energy. J. Power Sources.

[5] Charfi S., Atieh A., Chaabene M. (2016). Modeling and cost analysis for different PV/battery/diesel operating options driving a load in Tunisia, Jordan and KSA. Sustain. Cities Soc..

[6] Karami A., Basset P., Galayko D. Electrostatic vibration energy harvester using an electret-charged mems transducer with an unstable auto-synchronous conditioning circuit. Proceedings of the 15th International Conference on Micro and Nanotechnology for Power Generation and Energy Conversion Applications (PowerMEMS 2015).

[7] Xie X., Wang Q. (2015). A mathematical model for piezoelectric ring energy harvesting technology from vehicle tires. Int. J. Eng. Sci..

[8] Ng T.H., Liao W.H. (2016). Sensitivity analysis and energy harvesting for a self-powered piezoelectric sensor. J. Intell. Mater. Syst. Struct..

[9] Sharma P., Hajra S., Sahoo S., Rout P.K., Choudhary N.R.P. (2017). Structural and electrical characteristics of gallium modified PZT ceramics. Process. Appl. Ceram..

[10] Sahu M., Vivekananthan V., Hajra S., Abisegapriyan K.S., Raj N.P.M.J., Kim S.J. (2020). Synergetic enhancement of energy harvesting performance in triboelectric nanogenerator using ferroelectric polarization for self-powered IR signaling and body activity monitoring. J. Mater. Chem. A.

[11] Hosseini R., Hamedi M., Im J., Kim J., Dayou J. (2017). Analytical and experimental investigation of partially covered piezoelectric cantilever energy harvester. Int. J. Eng. Manuf..

[12] Lalthlamuana R., Talukdar S. (2016). Conditions of visibility of bridge natural frequency in vehicle vertical acceleration. Procedia Eng..

[13] Zhu Q., Yue J.Z., Liu W.Q., Wang X.D., Chen J., Hu G.D. (2017). Active vibration control for piezoelectricity cantilever beam: An adaptive feedforward control method. Smart Mater. Struct..

[14] Liu Y. (2015). Design Method of Piezoelectric Cantilever Beam in Bridge. Master’s Thesis.

[15] Xiong Y., Song F., Leng X. (2020). A piezoelectric cantilever-beam energy harvester (PCEH) with a rectangular hole in the metal substrate. Microsyst. Technol..

[16] Tong X., Song S., Wang L., Yang H. (2018). A preliminary research on wireless cantilever beam vibration sensor in bridge health monitoring. Front. Struct. Civ. Eng..

[17] Shi F., Tuo X., Yang S.X., Li H., Shi R. (2017). Multiple two-way time message exchange (TTME) time synchronization for bridge monitoring wireless sensor networks. Sensors.

[18] Kumalasari A.D., Tjondronegoro S. (2016). Design of structural health monitoring using wireless sensor network case study pasupati bridge. Appl. Mech. Mater..

[19] Li C., Cordovilla F., Ocana J L. (2018). Design optimization and fabrication of a novel structural piezoresistive pressure sensor for micro-pressure measurement. Solid State Electron..

[20] Dong Q., Wang J., Zhang X., Wang H., Jin X. (2019). Development of virtual load rating method for taxiway bridge under aircraft taxiing. KSCE J. Civ. Eng..

[21] Ye Z., Xiong H., Wang L. (2020). Collecting comprehensive traffic information using pavement vibration monitoring data. Comput. Aided Civ. Infrastruct. Eng..

[22] Yuan Y., Sun X., Liu Z., Li Y., Guan X. (2018). Approach of Personnel Location in Roadway Environment Based on Multi-sensor Fusion and Activity Classification. Comput. Netw..

[23] Aksel E., Jones J.L. (2010). Advances in lead-free piezoelectric materials for sensors and actuators. Sensors.

[24] Wang L., Tong X., Yang H., Wei Y., Miao Y. (2019). Design and analysis of a hollow triangular piezoelectric cantilever beam harvester for vibration energy collection. Int. J. Pavement Res. Technol..

